# Incorporating economic methods into Cochrane systematic reviews: case studies in brain tumour research

**DOI:** 10.1186/s13643-023-02254-w

**Published:** 2023-06-02

**Authors:** Ashleigh Kernohan, Tomos Robinson, Luke Vale

**Affiliations:** Health Economics Group, Population Health Science Institutes, Newcastle University, Baddiley Clark Building, Richardson Road, Newcastle upon Tyne, NE2 4AX UK

**Keywords:** Economic evaluation, Systematic review methods

## Abstract

**Background:**

Cochrane systematic reviews have established methods for identifying and critically appraising empirical evidence in health. In addition to evidence regarding the clinical effectiveness of interventions, the resource implications of such interventions can have a huge impact on a decision maker’s ability to adopt and implement them. In this paper, we present examples of the three approaches to include economic evidence in Cochrane reviews.

**Methods:**

The Cochrane Handbook presents three different methods of integrating economic evidence into reviews: the Brief Economic Commentary (BEC), the Integrated Full Systematic Review of Economic Evaluations (IFSREE) and using an Economic Decision Model. Using the examples from three different systematic reviews in the field of brain cancer, we utilised each method to address three different research questions. A BEC was utilised in a review that evaluates the long-term side effects of radiotherapy (with or without chemotherapy). An IFSREE was utilised in a review comparing different treatment strategies for newly diagnosed glioblastoma in the elderly. Finally, an economic model was included in a review assessing diagnostic test accuracy for tests of codeletion of chromosomal arms in people with glioma.

**Results:**

The BEC mirrored the results of the main review and found a paucity of quality evidence with regard to the side effects of radiotherapy in those with glioma. The IFSREE identified a single economic evaluation regarding glioblastoma in the elderly, but this study had a number of methodological issues. The economic model identified a number of potentially cost-effective strategies for tests for codeletion of chromosomal arms 1p and 19q in people with glioma.

**Conclusions:**

There are strengths and limitations of each approach for integrating economic evidence in Cochrane systematic reviews. The type of research question, resources available and study timeline should be considered when choosing which approach to use when integrating economic evidence.

## Background

Systematic reviews are an essential academic activity. With an estimated two and a half million scientific articles being published each year, the ability to reliably critique and synthesise this evidence is vital [1]. This is the goal of Cochrane (previously known as the ‘Cochrane Collaboration’), formed in 1993 to promote up to date systematic reviews of relevant evidence to inform health and care practice [2] using rigorous methods standards set out in the Cochrane Handbook [3]

While the effectiveness of health technology is an important factor for decision making, it is increasingly important to consider the resource implications of adopting of a new intervention and the impact it could have on health care systems. With increased pressures on the health care, providing decision makers with both the clinical and resource implications could allow for more efficient decision making, as all aspects of the intervention could be considered simultaneously. This will include summarising the data from randomised controlled trials and economic evaluations, which is an established method of comparing alternative courses of action in terms of their costs and consequences [4].

To provide decision makers with information about the cost effectiveness of a health technology, the recent Cochrane Handbook included a chapter focussing on how to include economic evidence alongside the effectiveness evidence [5]. Several methods are proposed in the chapter to incorporate economic evidence within an intervention review. These vary in the amount of researcher time and resources required, and as such, the approach must be chosen based on the specific research question being asked. These three methods were as follows:

1. A Brief Economic Commentary (BEC)
2. An Integrated Full Systematic Review of Economic Evaluations (IFSREE)
3. An economic decision model

In 2017, a collaboration between Cochrane, the National Cancer Research Institute (NCRI) and the National Institute for Health and Care Excellence (NICE) resulted in a programme of research to deliver a suite of eight systematic reviews in prioritised areas of brain tumour research [6]. For this particular suite of systematic reviews, the novel decision was made that for each review, a suitable economic method would also be incorporated to summarise the available economic evidence for each of the interventions that the review was investigating. As such, all three techniques were utilised within the context of this suite of research. The aim of this paper is to discuss the strengths and limitations of each approach for the inclusion of economic evidence in Cochrane Reviews, using three of the systematic reviews conducted as part of the suite of research as illustrative case studies.

### Brief Economic Commentary

The Brief Economic Commentary (BEC) was designed to summarise the existing economic evidence. This is a short summary of key economic evaluations relevant to the research question. As it is strictly only a summary of key aspects of the existing evidence, of the three approaches set out above it, both the least amount of experience with economics and the least amount of researcher time. This method was designed to be able to be carried out without requiring specialist input from health economists, beyond initial guidance and training in the method and procedures [5].

**Fig. 1:**
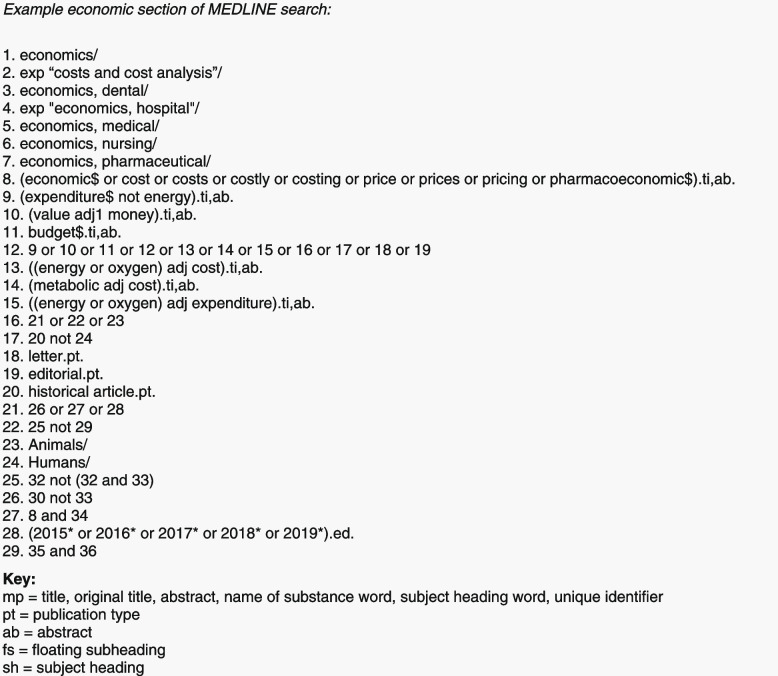
Example of the use of economic search terms for the specific economic search, from McBain et al. [6]

When including any economic evidence in a Cochrane review, the first stage is to include relevant economic information in the background section. This can include referring to previous studies which assess the costs of illness of the condition, costs of the intervention and relevant issues around cost-effectiveness (e.g., changes in the clinical area that could impact resource use). An additional economic search is also conducted which reported in the search section of the “Methods” section. To identify the intervention, the same search terms used in the main effects search are used. However, terms related to study design (e.g., a randomised trial) are replaced by those used to identify economic evaluation studies. An example of an economic search strategy is shown in Fig. 1, which is taken from the Cochrane Economics Methods chapter [5]. The economic background section and economic search stage is common to all three of the integration methods discussed in this paper.

There are two factors to consider when deciding about the inclusion of economic studies into a BEC. The first is the suitability of the interventions being evaluated. The inclusion criteria for the interventions should be the same as those for effectiveness review. This includes the population, intervention, comparison, and outcomes (PICO). The second consideration is the type of economic evaluation studies which can be included. The inclusion criteria for economic evaluations can include evaluations conducted alongside clinical studies (trial-based evaluations) and those which used existing literature to create an economic model (model-based evaluations). Other factors to be considered when considering studies for inclusion include outcome measures utilised such as clinical outcomes, quality-adjusted life years (QALYs) or monetary measures of benefit. If both interventions being analysed and the economic methods meet the inclusion criteria, then the study should be included.

For a BEC, the studies can be screened for inclusion by a single reviewer. Once suitable economic evaluations have been identified, the conclusions are reported as the author reports them. As such, the author’s own words should be used wherever possible. The key factors that should be reported in the BEC are the analytic framework, the perspective of the evaluation and the main items that were costed and the setting of the evaluation (country, currency, year) [5]. The resulting paragraph should summarise the author’s conclusion and any uncertainty around those conclusions. A key point that should be highlighted in the discussion is the lack of independent quality assessment. As such the author’s conclusions are presented at face value, it is important to state this explicitly either at the beginning or the end of the commentary, so that the readers of the review are aware of this.

#### BEC case study—taken from Lawrie et al. [7]

To understand the long-term side effects (neurocognitive or otherwise) of radiotherapy for those with glioma (with or without chemotherapy), a systematic review of studies comparing the side effects of different treatments was carried out as part of the previously described programme grant [7]. The review included randomised and non-randomised trials and controlled before and after studies (CBAS). The clinical component of the review concluded that there is some evidence of an increased risk of neurocognitive side effects in those who undergo radiography with a good prognosis, but the evidence is uncertain due to paucity of data and risk of bias.

To compliment the clinical component of the review, a BEC was included. The results of the BEC found no relevant economic evaluations with regard to the side effects of radiotherapy for those with glioma. This further highlighted a paucity of evidence of long-term side effects of radiotherapy in those with glioma. The review concludes that in addition to the necessity for high quality clinical studies, there is also a need for economic evaluation studies to understand the resource implication of any potential side effects from radiotherapy.

No economic evaluations were identified in this particular BEC; however, it was made clear that not we did not attempt to draw any firm or general conclusions regarding the relative costs or efficiency of studies due to the potential lack of quality appraisal of an identified evaluations. The BEC in this context provides a snapshot of the current economic literature without drawing firm conclusions regarding the cost-effectiveness of the strategies.

### Integrated Full Systematic Review of Economic Evaluations

Where firmer conclusions regard the cost-effectiveness evidence are necessary, an Integrated Full Review of Economic Evaluations (IFSREE) may be used instead of a BEC. This approach should be prioritised in reviews where the cost-effectiveness of the intervention is likely to be a key part of the decision of whether it is adopted or not. Like a BEC, relevant economic information should be included in the background section. The economic inclusion criteria should be specified, and a separate economic search should be carried out to identify relevant studies.

While a BEC can be screened by a single reviewer, an IFSREE must be screened by two reviewers independently, with a third reviewer acting as a mediator if necessary. This is because the IFSREE is reported in the results section of the review rather than the discussion. Once relevant studies have been chosen, the data should be extracted into a suitably designed data extraction template. Key information that should be extracted will include the type of evaluation, the analytical approach, the sources of the effectiveness data and the sources of the costs. An example of this kind of extraction table is shown in Table 1. It is also important to extract any relevant unit costs from included studies, as shown in Table 2.

**Table 1:** Data extraction table, from Hanna et al. [8]

**Data extraction table**					
**Author(s), year & title**	**Type of evaluations**	**Sources of effectiveness data**	**Sources of cost data**	**Sources of outcome valuations**	**Analytical approach**
**Ghosh et al. **[9] Improved cost-effectiveness of short-course radiotherapy in elderly and/or frail patients with glioblastoma	Cost effectiveness analysis Cost utility analysis	Effectiveness data was taken from a phase III randomised trial.	Direct unit medical costs were collected from each country participating in the trial (from 2015).	Outcomes were presented overall survival (OS) and progression free survival (PFS). The outcomes were also presented as quality adjusted life years (QALYs). Preference values were taken from European Organization for Research and Treatment of Cancer Quality of Life Questionnaire (EORTC QLQ-C30). The scores were mapped onto the EQ-5D questionnaire from which utility values were derived. The values were taken from three sources; Kontodimopoulos 2009, Kim 2012 and McKenzie 2009. The QALY calculation based on the assumption that the patient on treatment is to live for 4 months.	Restricted mean overall survival (RMOS) Incremental cost effectiveness ratio (ICER).

**Table 2:** Cost data extraction table, from Hanna et al. [8]

**Component**	**Study**	**Country**	**Estimated costs of resources used**	**Source**	**Currency**	**Average number of resources used** **Arm 1 / Arm 2**		**Source**
Dexamethasone 4 mg tablet	Ghosh et al*.* [9]	Belarus	0.27	Not reported	US dollars (conversion not reported)	44	16	Trial dataset
		Brazil (Porto Alegre)	0.06	Not reported	US dollars (conversion not reported)	44	60	Trial dataset
		Georgia	0.3	Not reported	US dollars (conversion not reported)	18	4	Trial dataset
		India	0.01	Not reported	US dollars (conversion not reported)	32	24	Trial dataset
		Poland	1.36	Not reported	US dollars (conversion not reported)	12	8	Trial dataset

Once data has been extracted from the relevant studies, a quality appraisal of the economic evidence is necessary. The first stage of assessing the quality of the study is assessing the quality of the underpinning source of evidence. If the economic evaluation is based upon a randomised controlled trial then this should be carried out using the Cochrane Risk of Bias Tool 2 (ROB2) [10]. Logically, if an economic evaluation is based upon a randomised trial then a risk of bias assessment should consider just the economic outcomes (when the ROB2 tool is used as is currently recommended by the Cochrane Handbook). The next part of the quality assessment is to assess the quality of the economic methods of the evaluation, which should be assessed with one of two separate tools. The recommended quality checklists for within trial evaluations are the Consolidated Health Economic Evaluation Reporting Standards (CHEERS) checklist [11] or the Consensus Health Economic Criteria (CHEC) list [12]. For model-based evaluations, the checklists that are recommended are the CHEERS checklist and the Combined NICE ‘Study limitations’ checklist [13]. The studies should also make reference to the Phillips checklist, which focuses on methodological quality [14]. The results of these checklists should be reported in the text, and the full checklists should be included in the appendices.

Once the data has been extracted and the quality of the studies has been assessed, the data must then be synthesised. Research into the meta-analysis of economic evaluations is ongoing, but at present, it is recommended to carry out a narrative synthesis. For the narrative synthesis, the recommended data is presented in key tables and patterns in observed effect sizes should be observed. In addition, any conflicting evidence between studies and settings should be discussed, including possible explanations for these differences.

#### IFSREE case study—taken from Hanna et al. [8]

A further review within the Cochrane programme grant assessed different treatment strategies for newly diagnosed glioblastoma in the elderly [8]. The aim of this review was to find the most effective and best-tolerated approaches for elderly individuals with newly diagnosed glioblastoma. Randomised controlled trials including participants who were over 65 with newly diagnosed glioblastoma were included. The review found evidence to support the use of chemoradiotherapy (CRT) compared with radiotherapy (RT). The review also found that systemic anti-cancer treatments temozolomide (TMZ) and bevacizumab BEV carry a higher risk of severe haematological and thromboembolic events and that there is probably very limited evidence for the use of BEV in elderly patients outside a clinical trial setting. This study included an IFSREE which contained one study identified for inclusion [9]. The data extraction tables are shown in Tables 1 and 2.

The single economic evaluation study which was identified was based on a trial and compared the short course to standard radiotherapy in elderly patients [9]. The evaluation reported that the short course radiotherapy intervention was cost-effective compared to the standard radiotherapy intervention. However, the results of the quality assessment of the study found that although the source of the effectiveness data had a low risk of bias, there were a number of issues with the economic evidence. It was noted the methods which were presented in the paper could not be replicated by the authors. As such, the conclusion was that there was currently a paucity of high-quality evidence for interventions for newly diagnosed glioblastoma in the elderly.

### Economic decision model

In addition to the IFSREE, it is possible to build upon the full review of evidence and include an economic decision model in a Cochrane review. A decision model can be described as using mathematical relationships to define a series of possible consequences that would flow from a series of alternative options being evaluated [15]. All economic decision models involve several stages including designing the model structure, identifying the necessary data to populate the model and running the different analyses, with the specific design of the model reflecting the decision problem at hand. More information regarding the different approaches to health economic decision modelling can be found in Briggs et al. and the ISPOR good modelling practice guidelines [15, 16]. The use of an economic model will require the use of the IFSREE approach as the first stage of the economic component of the review. This will make it possible to collate the evidence as detailed data from the data extraction phase of the review will be used to parameterise the model. For example, the unit costs extracted from papers can represent a range of unit costs to be used in a decision model or relative effect size estimated in an intervention review of sensitivity and specificity values from a diagnostic review may be used in a diagnostic model. It may also be necessary to carry out a sensitivity analysis to assess how robust the conclusions of the model and explore the uncertainty in the conclusions. This approach allows the extracted data to be used to answer a specific question relevant to the review.

#### Economic decision model case study—taken from McAleenan et al. [17]

A review to assess diagnostic test accuracy and cost-effectiveness of tests for codeletion of chromosomal arms 1p and 19q in people with glioma was included as part of the programme grant [17]. As such, this review assessed the diagnostic test accuracy (in terms of sensitivity and specificity) for each of these tests. Cross-sectional studies which assessed 1p/19q status using two or more tests were considered for inclusion, and a number of different tests were included in the review the full details of which are detailed in the published review [17]. The results of the clinical part of the review concluded that although current guidelines recommend that 1p/19q-co-deletion should be evaluated to support a diagnosis of oligodendroglioma, there is no consensus as to the best approach. Potentially promising testing strategies include next-generation sequencing (NGS) and single-nucleotide polymorphism arrays (SNP) but further research is needed.

**Fig. 2:**
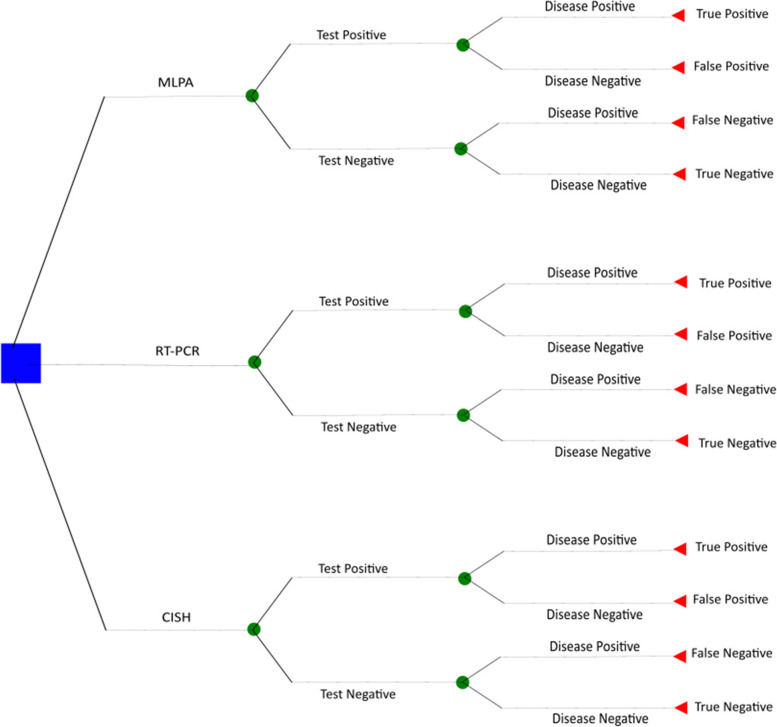
Example of decision tree model. Figure from McAleenan et al. [17]

The IFSREE found no existing economic evaluations that assessed the cost-effectiveness of tests of codeletion of chromosomal arms 1p and 19q in people with glioma. To assess the costs and benefits of different testing strategies using existing data, a simple decision tree model was designed (this can be seen in Fig. 2). Intervention costs were derived from both an expert opinion from within the Newcastle upon Tyne Hospitals NHS Foundation Trust based on internal costings and existing literature. The results of the economic model implied that potential strategies which could be cost-effective were MLPA, RT-PCR, CISH, SNP Array and NGS. Taking FISH as a reference standard and focusing on the ability to make a correct diagnosis, all the tests except MLPA and CISH were found to be an inefficient efficient use of resources. When PCR-based LOH was used as the reference standard, MLPA was found to be the most cost-effective strategy. Sensitivity analysis showed the results show that cost-effectiveness is sensitive to both the choice of the reference standard and the decision maker’s willingness to pay for the additional benefit.

With regards to this model, it should be noted that due to the paucity of evidence that was found, the conclusions are based on a small number of studies with a small number of overall participants. Another consideration of this model is that although the testing costs were included, subsequent costs such as the resulting complications of a false negative test were not fully explored. These limitations were discussed in the discussion section of the original review. This model highlighted promising strategies in terms of cost-effectiveness for future research.

## Discussion

In this study, we have described and presented three different methods for including economic methods in Cochrane systematic reviews. The examples used in this study specifically relate to brain cancer; however, these methods have also be used in a number of other clinical areas to incorporate economic outcomes into the results, including reviews related to eye care and incontinence [18, 19]. The advantages and disadvantages of the three methods explored in this paper are shown in Table 3.

**Table 3:** Advantages and disadvantages of an economic approach

**Method**	**Advantages**	**Disadvantages**
Brief Economic Commentary (BEC)	Can be carried out by single health economics reviewer. Studies can be screened and data can be extracted by a single reviewer.	No formal critical appraisal. Conclusions presented at face value in authors of the economic evaluation’s own words.
Integrated Full Systematic Review of Economic Evaluations (IFSREE)	Full critical appraisal given to each included study. Data extracted more granularly. More robust conclusion can be drawn	A minimum of two economic reviewers are needed to carry out the IFSREE. More analysis time needed to carry out extraction and quality assessment stages.
Economic decision model	Can draw primary conclusions about decision problem using data from economic and clinical review about decision problem.	A minimum of two economic reviewers are needed to carry out the IFSREE. Can be time consuming depending on complexity of model structure. Can be limited by data availability to populate model.

The first method, the BEC, has the distinct advantage of not requiring separate reviewers to carry out. Indeed, it is possible for a reviewer who is less familiar with economics to carry out (ideally under the supervision of someone who is familiar with economics, but this is not essential). The BEC does not also require the double screening and data extraction stages required of other methods of including economics in systematic reviews. This makes it much more accessible to a team of reviewers as it requires less resources. The principal disadvantage of the BEC approach is the lack of quality appraisal of any included studies. Although this must be stated plainly in the text, there is still a risk that the conclusion of an economic study author being taken at face value even if there are methodological issues with the study. This can be clearly illustrated when considering the IFSREE example. Although one study was identified that was relevant to the topic area, the quality assessment revealed a number of methodological issues with the economic methods adopted and as such it could not be taken to be a reliable source of evidence. Had a BEC approach been used in this instance then the methodological issues may not have been identified and key information about the paper may not have been included. The disadvantage of the IFSREE approach is that it is much more resource intensive than the BEC. At least two reviewers who are familiar with economic methods are required to screen, extract and synthesise the data which may be difficult for reviewers who have no previous experience including economics in their reviews. Ultimately a greater collaboration between those who are involved in systematic review, both those with a clinical interest and those who are driving methodological development would benefit from greater collaboration with health economists.

The use of an economic model in a Cochrane Review has the potential to use the data which is extracted as part of the IFSREE. This approach will allow for primary data analysis relevant to the decision problem to be included in a review and has the potential to be a useful and important tool in Cochrane reviews. At present, there are few economic decision models which have been incorporated into Cochrane reviews, but there is greater scope for future inclusion. One limitation of the inclusion of an economic model in a review is that the quality of the model will critically depend on the quality of the clinical and economic evidence. As shown in the case study presented, if there is a paucity of clinical or economic evidence, it will be difficult to populate the populate an economic decision model. In addition to the limitations with populating the model, there may also be a limitation with regard to the generalisability of both studies that populate the model and the findings of a model overall. If there are limitations in the available data to parameterise the decision model, there are other ways that parameters can be populated. These include, but are not limited to, utilising clinical expert opinion or using estimates from adjacent studies. However, it should be noted that the robustness of the conclusion of the model will in part be dependent on reliable parameters that are used to populate it.

Economic evaluations are often carried out with reference to a particular health care system. As such, the particular applicability of the number of resources and the amount that these costs may differ from country to country even when adjusting for currency differences and inflation. Future research could address issues relating to the best methods to handle missing data in models in Cochrane reviews. A further limitation of the inclusion of an economic model is the resources required compared to the other approaches. Researcher time is needed to design, to model and to gather data required to parametrise the model as well as the analysis and reporting. The additional work required to complete the design and analysis of the decision model may impact the delivery of the systematic review.

## Conclusion

In summary, there are several different ways that economic methods can be included in Cochrane systematic reviews. The use of a BEC can be a useful introduction to the inclusion of economic evidence and give a flavour of the existing literature. However, a BEC cannot be used to derive any firm conclusions about the cost-effectiveness of the intervention. The IFSREE is a more detailed approach and is such more resource intensive but will have a greater ability to draw conclusions regarding the cost-effectiveness of the intervention being assessed. Finally, an economic model is a method to answer a specific decision question based on the data, which is extracted as part of the review, but for the model to be reliable a good level of existing evidence is required for parameterisation. The choice of method should be guided by the specific research question and the resources available. The use of an integrated economic component allows for information regarding the resource implications of the inclusion of health technology to be made synthesised and made available to decision makers. Broadening the use of economics within systematic reviews and Cochrane Reviews will allow more information to be considered when considering policy and practice in health care.

## References

[1] Ware, M. and M. Mave, The STM report: an overview of scientific and scholarly journal publishing., T.A.M.P. International Association Of Scientific, Editor. 2015. The Hague: International Association of Scientific, Technical and Medical Publishers; 2018.

[2] Cochrane, A., Efficiancy and effectiveness: random reflections on health services, ed. T.N.P.H. Trust. London; Nuffield Provincial Hospitals Trust. 1972.

[3] Higgins, J., et al., Cochrane Handbook for Systematic Reviews of Interventions version 6.2 (updated February 2021), A.f. www.training.cochrane.org/handbook., Editor. 2021, Cochrane.

[4] Drummond MF, Methods for the economic evaluation of health care programmes.  (2015). Oxford.

[5] Aluko, P., et al., Chapter 20: economic evidence., in Cochrane Handbook for Systematic Reviews of Interventions version 6.2 (updated February 2021). , J. Higgins, et al., Editors. 2020, Available from www.training.cochrane.org/handbook.

[6] McBain C, Lawrie TA, Rogozińska E, Kernohan A, Robinson T, Jefferies S. Treatment options for progression or recurrence of glioblastoma: a network meta‐analysis. Cochrane Database Syst Rev. 2021;(1):CD013579. 10.1002/14651858.CD013579.pub2. Accessed 01 June 2023. 10.1002/14651858.CD013579.pub2 PMC8121043 34559423

[7] Lawrie TA (2019). Long-term neurocognitive and other side effects of radiotherapy, with or without chemotherapy, for glioma. Cochrane Database Syst Rev.

[8] Hanna C (2020). Treatment of newly diagnosed glioblastoma in the elderly: a network meta-analysis. Cochrane Database Syst Rev.

[9] Ghosh S, et al. Improved cost-effectiveness of short-course radiotherapy in elderly and/or frail patients with glioblastoma. Radiother Oncol. 2018;127(1):114–20. 10.1016/j.radonc.2018.01.017 29452901

[10] Higgins JPT, Thomas J, Chandler J, Cumpston M, Li T, Page MJ, Welch VA (editors). Cochrane Handbook for Systematic Reviews of Interventions version 6.3 (updated February 2022). Cochrane, 2022. Available from www.training.cochrane.org/handbook.

[11] Husereau D (2022). Consolidated health economic evaluation reporting standards 2022 (CHEERS 2022) statement: updated reporting guidance for health economic evaluations. Int J Technol Assess Health Care.

[12] Evers S (2005). Criteria list for assessment of methodological quality of economic evaluations: consensus on health economic criteria. Int J Technol Assess Health Care.

[13] National Institute of Health and Care Excellence, Guide to the methods of technology appraisal in Process and methods, N.I.o.H.a.C. Excellence, Editor. 2013. 27905712

[14] Philips Z (2004). Review of guidelines for good practice in decision-analytic modelling in health technology assessment. Health Technol Assess.

[15] Briggs A, Sculpher M, Claxton K (2006). Decision modelling for health economic evaluation. Handbooks in Health Economic Evaluation Vol. Volume 1.

[16] Caro JJ (2012). Modeling good research practices–overview: a report of the ISPOR-SMDM Modeling Good Research Practices Task Force–1. Value Health.

[17] McAleenan A (2022). Diagnostic test accuracy and cost-effectiveness of tests for codeletion of chromosomal arms 1p and 19q in people with glioma. Cochrane Database Syst Rev.

[18] Lawrenson JG (2023). Interventions for myopia control in children: a living systematic review and network meta-analysis. Cochrane Database Syst Rev.

[19] Eliezer D, Deshpande AV, Starkey MR, Samnakay N, Oldmeadow C, Kernohan A. Alpha blockers for treating functional daytime urinary incontinence in children. Cochrane Database Syst Rev. 2019;(4):CD013313. 10.1002/14651858.CD013313. Accessed 01 June 2023.

